# The Future of Bronchopulmonary Dysplasia: Emerging Pathophysiological Concepts and Potential New Avenues of Treatment

**DOI:** 10.3389/fmed.2017.00061

**Published:** 2017-05-22

**Authors:** Jennifer J. P. Collins, Dick Tibboel, Ismé M. de Kleer, Irwin K. M. Reiss, Robbert J. Rottier

**Affiliations:** ^1^Department of Pediatric Surgery, Sophia Children’s Hospital, Erasmus University Medical Centre, Rotterdam, Netherlands; ^2^Division of Pediatric Pulmonology, Department of Pediatrics, Sophia Children’s Hospital, Erasmus University Medical Centre, Rotterdam, Netherlands; ^3^Division of Neonatology, Department of Pediatrics, Sophia Children’s Hospital, Erasmus University Medical Centre, Rotterdam, Netherlands

**Keywords:** bronchopulmonary dysplasia, chronic lung disease of prematurity, respiratory distress syndrome, preterm birth, lung development, chronic lung disease

## Abstract

Yearly more than 15 million babies are born premature (<37 weeks gestational age), accounting for more than 1 in 10 births worldwide. Lung injury caused by maternal chorioamnionitis or preeclampsia, postnatal ventilation, hyperoxia, or inflammation can lead to the development of bronchopulmonary dysplasia (BPD), one of the most common adverse outcomes in these preterm neonates. BPD patients have an arrest in alveolar and microvascular development and more frequently develop asthma and early-onset emphysema as they age. Understanding how the alveoli develop, and repair, and regenerate after injury is critical for the development of therapies, as unfortunately there is still no cure for BPD. In this review, we aim to provide an overview of emerging new concepts in the understanding of perinatal lung development and injury from a molecular and cellular point of view and how this is paving the way for new therapeutic options to prevent or treat BPD, as well as a reflection on current treatment procedures.

## Introduction

Yearly over 15 million babies are born premature (<37 weeks gestational age), accounting for more than 1 in 10 births worldwide, of which approximately 2.4 million babies are born before 32 weeks of postmenstrual age (PMA) (1). Bronchopulmonary dysplasia (BPD) is the most common adverse outcome in very preterm neonates with an incidence of 5–68%, depending on the cohort and definition used, which increases significantly with declining gestational age (2, 3). BPD develops as a result of lung injury caused by maternal pre-eclampsia, chorioamnionitis, postnatal ventilation, hyperoxia, and/or inflammation, leading to an arrest in alveolar and microvascular development and pulmonary hypertension, although the relative contribution of the different pathogenic factors for the individual patient is hard to identify (4). Originally, BPD (“old” BPD) was defined based on lung injury resulting from mechanical ventilation and oxygen supplementation, and was seen mostly in premature infants born at 26–30 weeks PMA (5–7). The introduction of major interventions such as maternal corticosteroids (8, 9) and surfactant replacement therapy (10–12) resulted in a changed disease phenotype that was seen in preterm infants that could survive at younger gestational ages (24 to 26 weeks PMA). As a result, “new” BPD, defined as the requirement of supplemental oxygen at 36 weeks PMA or treatment with supplemental oxygen for more than 28 days (4), was characterized based on impaired alveolar and capillary development of the immature lungs (13). It is now becoming clear that BPD survivors continue to have respiratory morbidity after they leave the neonatal intensive care unit (NICU) [see comprehensive review by Islam et al. (14)], underlining that BPD really is a disease of disrupted lung development. Understanding how the alveoli and underlying capillary network develop and how these mechanisms are disrupted in BPD is critical for developing efficient therapies, which currently are lacking. Moreover, the nature of lung injury and consequently BPD is perpetually changing as treatment strategies evolve in an attempt to prevent injury to the premature lungs. Combined with increasing insight into the pathophysiology of BPD, this has started a discussion on yet a newer definition of what BPD is, basing it more on biomarkers, pulmonary hypertension and the underlying vascular basis of BPD (15–17). In this review, we provide an overview of emerging new pathophysiological concepts in the understanding of perinatal lung development and injury from a molecular and cellular point of view and how this is paving the way for new therapeutic options to prevent or treat BPD, as well as a reflection on how this compares with current treatment procedures.

### Overview of Lung Development

To understand BPD pathophysiology, it is important to understand how the lung normally develops. Despite the large body of knowledge concerning the morphogenesis of the lung (18, 19), research on the intercellular communications that regulate growth, migration, and differentiation during lung development is still unfolding. Among the best characterized growth factors and their signaling components in early lung development are fibroblast growth factor (FGF), transforming growth factor β (TGFβ), bone morphogenetic protein (BMP), sonic hedgehog (SHH), wingless-type MMTV integration site family (WNT), vascular endothelial growth factor (VEGF), and retinoic acid signaling pathways [reviewed by Hogan and Morrissey (20) and Kool et al. (21)]. Far less is known about the molecular and cellular processes that direct saccular and alveolar development, the very stages that are clinically relevant after preterm birth and BPD pathogenesis. VEGF, which is expressed by alveolar epithelial type II cells in response to hypoxia-induced factor (HIF), is crucial in directing pulmonary microvascular development and alveolar development (22). Moreover, VEGF plays an important role in BPD pathogenesis as BPD patients express little or no VEGF in their lung epithelium, and lack expression of VEGF receptors in pulmonary microvascular endothelium (23). Multiple studies have demonstrated that platelet derived growth factor (PDGF) and FGF signaling is crucial for myofibroblast differentiation and subsequent onset of secondary septation (24–29). WNT, BMP, and TGFβ signaling components have also been implicated to play a role in fibroblast differentiation during alveolarization (30–32). Additionally, correct deposition of extracellular matrix (ECM) proteins by myofibroblasts, like elastin and collagen, plays a crucial role during secondary septation (33, 34). These and other ECM components may exert their role in lung development by functioning as a scaffold for the growth factors to coordinate the growth interactions of cells (35).

## BPD in 2017

### Current Understanding of Perinatal Risk Factors

**Figure 1:**
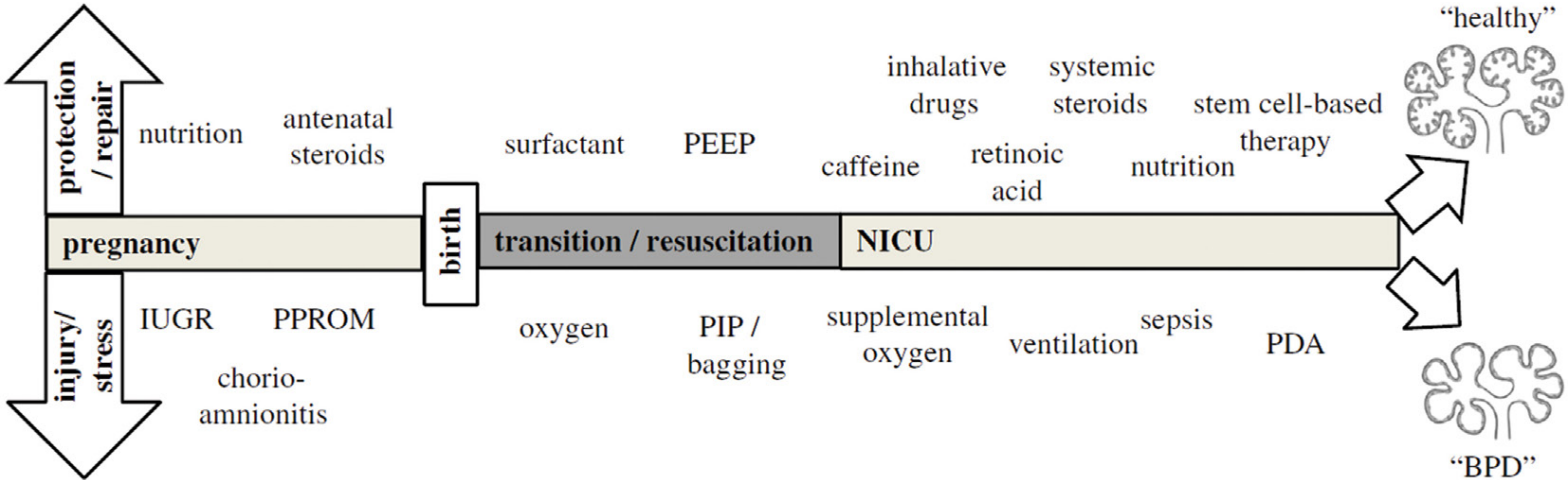
**The pathogenesis of bronchopulmonary dysplasia (BPD) is highly multifactorial in nature, with a wide variety of pre- and postnatal exposures influencing lung development**. Depending on the timing and combinations of exposures, BPD likely exists of multiple different pathophysiologies that manifest themselves in a similar way clinically. The top arrow represents exposures that may to a certain extent protect from BPD pathogenesis and promote repair, while the bottom arrow indicates exposures that injure the preterm lung and contribute to BPD pathogenesis. Figure reprinted from Hütten et al., originally published by Springer (36).

Because BPD is still very much a functional diagnosis, which is made when preterm infants have already been exposed to a wide variety of perinatal stressors [Figure 1; (36)], it is hard to pinpoint exactly which exposure is more detrimental for lung development. Most of these insights have been obtained through decades of work on animal models [reviewed by Jobe (37)] and correlations found through epidemiological research. Already before preterm birth, intrauterine conditions can have a profound impact on lung development and susceptibility to BPD. Risk factors established by statistical correlation are first and foremost maternal risk factors associated with preterm birth, such as smoking and socioeconomic background (38). Intrauterine growth restriction increases the risk of BPD threefold in infants born before 29 weeks (2, 39), while chorioamnionitis and pre-eclampsia trigger the release of cytokines and growth factors that directly inhibit alveolar and microvascular development of the fetal lungs (2, 36, 40). Placental abnormalities, such as gestational hypertension, pre-eclampsia, and eclampsia, are emerging as an important antenatal risk factor for BPD. A French prospective cohort study found that placenta-mediated pregnancy complications with fetal consequences are associated with moderate to severe BPD in very preterm infants (41). The maternal administration of corticosteroids prior to preterm birth leads to thinning of the primary septa, which narrows the air blood barrier, stimulates the production of surfactant, which stabilizes the alveolar sacs and prevents collapse after exhalation, and stimulates the clearance of fetal lung fluid (42). Although this accelerated development improves neonatal outcome and survival of the infant, antenatal corticosteroids have the unwanted side effect of inhibiting secondary septation and impairing microvasculature development (28, 43–45).

Postnatally, inflammation is also considered to be an important risk factor for the development of BPD [reviewed in Ref (46)], either as a result of lung injury caused by invasive mechanical ventilation and supplemental oxygen or in the form of sepsis. Due to their lung immaturity and apnea of prematurity, preterm infants are also frequently exposed to hypoxia, which just like hyperoxia leads to impaired alveolar and microvascular development (47). Recently, the presence of oxygen-sensitive intrapulmonary bronchopulmonary anastomoses (IBA) was discovered in preterm infants with BPD and other infants with chronic lung diseases, which may stay patent in the setting of persistent hypoxia (16, 48–52). Thus, IBA may in itself lead to persistent hypoxemia and contribute to the pulmonary hypertension that is often seen in conjunction with BPD, and could therefore be a significant risk factor for BPD (16). Considering that not all infants that are born very or extremely preterm go on to develop BPD, multiple pre- and/or postnatal hits are probably needed for lung development to be significantly affected, especially since the incidence of BPD has not decreased despite advances in neonatal care (2).

### Current Treatment Procedures

In this complex multifactorial setting, current therapies are aimed to not only support the survival of the preterm infant, but also to limit or prevent further damage as much as possible [see review by Jain and Bancalari (53)]. In this regard, the most direct approach is to prevent the need for aggressive, prolonged invasive ventilation. The first treatment of choice to prevent respiratory distress syndrome (RDS) is still antenatal maternal corticosteroid administration, followed by prophylactic surfactant therapy through endotracheal bolus administration after birth. The maternal administration of a single or repeated intramuscular injection of betamethasone or dexamethasone within a time window of 24 h to 7 days prior to preterm birth can significantly increase survival of the preterm infant and decrease the incidence and severity of RDS (9, 54). However, there is no consensus yet on how the use of antenatal steroids can be optimized by improving the timing of administration and dosing (42). Similarly, there is discussion as to whether surfactant therapy should be prophylactic or only selectively administered upon diagnosed RDS, as a result of the increased use of non-invasive ventilation methods such as nasal continuous positive airway pressure (CPAP) (53, 55). Without the application of routine CPAP, prophylactic surfactant treatment reduces neonatal mortality. However, the routine application of CPAP reduces the risk of BPD and neonatal death, and in these infants selective administration of surfactant is more beneficial (55). The INSURE method (intubate-surfactant-extubate to CPAP) is therefore now the recommended technique to avoid lung injury (56).

An alternative method of surfactant administration that builds on this is less invasive surfactant administration (LISA), which circumvents the need of endotracheal intubation and mechanical ventilation all together while improving pulmonary outcome in extreme premature infants (57–59). A more high-tech approach that is now being tested in the NICU is surfactant administration through aerosolization, nebulization, or atomization (60–67). It has proven technically challenging to achieve sufficient delivery of surfactant in the distal lung compared to bolus administration of surfactant, although the recent development of vibrating membrane nebulizers seems promising (67). Switching from animal-derived surfactants to new generation synthetic surfactants, which are more resistant to inactivation and even anti-inflammatory in cell culture and animal studies, may be another step forward (11, 68–75). Several clinical trials are testing two promising synthetic surfactants to combat RDS in the NICU. A multicenter phase 2 study is comparing the safety and efficacy of CHF5633, a synthetic surfactant with surfactant protein (SP)-B and SP-C analogs, with poractant alfa in preterm infants with RDS (ClinicalTrials.gov identifier NCT02452476). In addition, two multicenter phase 2 studies are assessing the safety and efficacy of aerosolized lucinactant (also known as KL4 surfactant, Aerosurf, and Surfaxin) in preterm neonates 26 to 32 weeks PMA receiving nasal CPAP (ClinicalTrials.gov identifiers NCT02636868 and NCT02528318). Optimizing ventilation strategies and surfactant therapy are therefore seen as the most easily achievable targets in the prevention of BPD.

Besides ventilation strategies, surfactant therapy and corticosteroids, there are a few therapies that have a profound effect in the prevention of BPD. Prophylactic caffeine therapy is recommended to counter apnea of prematurity and is now common practice after it was shown to be effective in reducing BPD and subsequent neurodisability (56, 76–78). The protective effect of caffeine therapy appears greater when given earlier rather than later, although there is still discussion among experts as early therapy is also associated with slightly greater mortality in some studies (79–81). This effect has been attributed to infants receiving earlier extubation and subsequently shorter mechanical ventilation times, alleviating the injury burden on the developing premature lung (76, 79). Multiple recent animal studies have attempted to elucidate whether caffeine itself can promote or protect alveolar development directly, with mixed results. Using the hyperoxia model of experimental BPD, caffeine could protect against alveolar simplification and inflammation in rats (82, 83) and rabbits (84), but not in mice (85, 86). Potential mechanisms include its abilities to amplify glucocorticoid-mediated SP-B expression in alveolar type 2 cells (87, 88), to modulate connective tissue growth factor (CTGF) expression (89) and TGFβ pathway members (85), and to attenuate endoplasmic reticulum (ER) stress (82). Conflictingly, both up- and downregulation of alveolar apoptosis has been reported (82, 86). Caffeine is however primarily known as a methylxanthine, which is a non-selective phosphodiesterase (PDE) inhibitor (78). PDE inhibitors have potent immunomodulatory and vascular effects and are therefore still interesting targets for neonatal intensive care medicine. Animal studies using the neonatal rodent hyperoxia model of experimental BPD have shown promise for non-selective PDE inhibitor pentoxyfilline (90), PDE4 inhibitors rolipram, piclamilast, and cilomilast (91–93), and PDE5 inhibitor sildenafil (94), which were able to ameliorate pulmonary inflammation and hypertension and improve lung alveolarization. Inhaled nitric oxide (iNO) therapy, which has a complementary mode of action to PDE inhibitors by boosting cyclic guanosine monophosphate (cGMP) (95), has long been the subject of clinical trials after promising results in animal models of BPD. Although iNO decreases inflammatory mediators in tracheal aspirates of treated preterm infants (96), systematic reviews show no protective effect in the development of BPD (97). Interestingly, iNO therapy was effective in reducing BPD incidence when combined with vitamin A therapy (98). Supplementation with vitamin A improved alveolarization in neonatal rats and lambs (99, 100), while in clinical studies, supplementation with vitamin A in preterm infants significantly reduced the risk of BPD (101–103). Unfortunately, these studies have not lead to the adoption of vitamin A supplementation in clinical practice, as the treatment benefits were deemed too small and the intramuscular route of administration too cumbersome in tiny preterm infants (104, 105). Other administration routes must be investigated for these promising therapies to become commonplace in the clinic.

For all currently used therapies, there is still ground to be gained through clinical trials and evidence-based medicine to ascertain optimal dosing, timing, and administration methods for maximum efficiency. It is essential that risk stratification takes place within the trial design to identify the real potential advantage of the different interventions. Despite all efforts at reducing lung injury through current treatment procedures, the incidence of BPD has remained stable over the past two decades (2). This is in part explained by the increased survival of extremely preterm infants born between 22 and 26 weeks PMA but probably also reflects the highly multifactorial nature of BPD. Prematurity is often not the first complication leading to BPD pathogenesis, as infants have already been exposed to a disadvantageous intrauterine environment, either through severe intrauterine growth restriction resulting from severe pre-eclampsia or chorioamnionitis. This is then followed by various exposures and comorbidities in the NICU, which in a substantial portion of these extreme premature infants leads to BPD with a similar phenotype, even though the underlying pathogenesis might have been quite different. It should not be forgotten that an astonishing portion of these infants does not go on to develop BPD, despite experiencing similar exposures. A better understanding of the pathophysiology leading to BPD is therefore crucial to create a better tailored treatment regimen for premature infants.

## Current Understanding of BPD Pathophysiology, New Pathophysiological Concepts, and Potential Therapies

**Figure 2:**
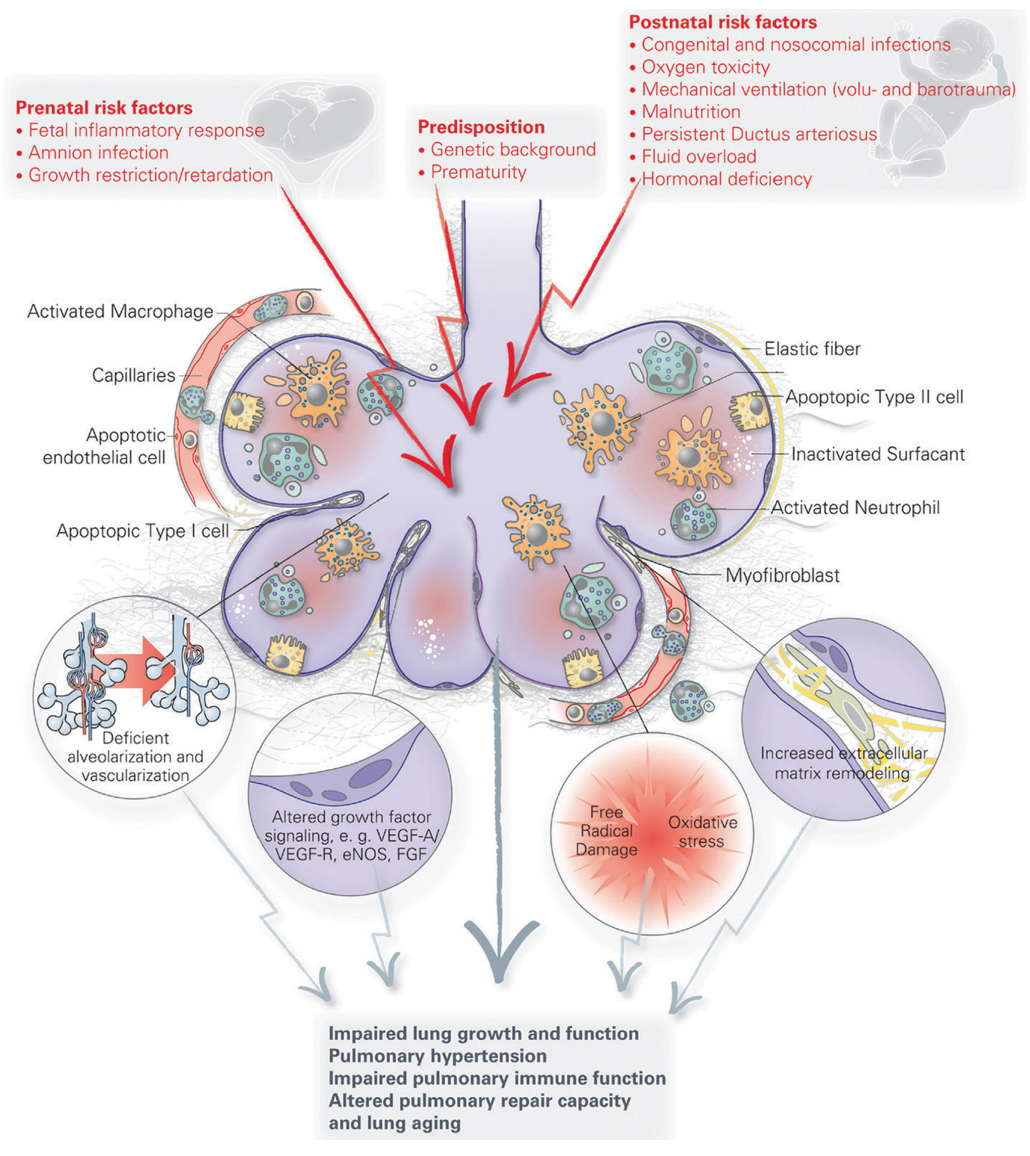
**A schematic overview of the pathophysiology of bronchopulmonary dysplasia (BPD)**. Pre- and postnatal risk factors lead to lung injury, resulting in apoptosis of distal lung cells, inflammation, extracellular matrix remodeling and altered growth factor signaling. These have long term effects on lung growth and function, including vascular and immune function, resulting in an increased disposition for chronic lung disorders. Figure reprinted from Hilgendorff and O’Reilly, originally published by Frontiers in Medicine (107).

Infants at greatest risk of developing BPD are born when their developing lungs are still transitioning from the canalicular to saccular phase. Given the complexity of lung development and the wide variety of perinatal insults leading to BPD, there is likely no single pathophysiology of BPD. Because of a paucity of histopathological data from preterm infants and BPD patients, our current understanding of BPD pathophysiology has mostly been generated from various small and large animal models looking at the effect of perinatal inflammation, oxygen toxicity, and mechanical ventilation on lung development [reviewed by Jobe (37)]. Although these simplified animal models of BPD only approximate the actual disease in humans, they have helped us immensely to better understand the pathophysiology of BPD. A number of recent reviews have generated a detailed overview of the various pathophysiological mechanisms implicated in BPD that have been uncovered through these models [see review by Niedermaier and Hilgendorff (106) and Hilgendorff and O’Reilly (107)], focusing on the role of perinatal infection and inflammation (46, 108, 109), pulmonary vascular development (17), the mesenchyme (110), the extracellular matrix (111), and oxygen (112) [Figure 2 (107)]. For the remainder of this review, we will highlight new pathophysiological concepts that are promising avenues for potential future therapies for BPD. Because of the inherent intertwinement of the pathophysiological mechanisms and potential therapies, we have chosen to present these side by side for each pathophysiological concept.

### Stem Cells in Development and for Therapy of BPD

In the past decade, the field of stem cell biology has advanced significantly, especially with respect to tissue resident stem cells in development and repair. A wide variety of lung epithelial stem/progenitor cells has been described but also multipotent mesenchymal stromal cells (MSCs) and endothelial colony forming cells (ECFCs) [reviewed in Ref (113)]. In the developing lung, where an extensive microvasculature is crucial for lung function, resident lung MSCs (L-MSCs) are a heterogeneous progenitor population, which orchestrate the formation of the alveolar microvasculature, repair/regeneration, and tissue maintenance [reviewed in Ref (114, 115)]. Already at the beginning of lung budding, a multipotent cardiopulmonary mesoderm progenitor has been described, based on expression of Wnt2, Gli1 and Isl1, giving rise to pulmonary vascular and airway smooth muscle, proximal vascular endothelium and pericyte-like cells (116). During pseudoglandular lung development early Tbx4^+^ multipotent MSCs give rise to a wide variety of distinct mesenchymal cell populations including airway and vascular smooth muscle and early fibroblast-like cells (117), reminiscent of quintipotential MSCs in bone marrow (118). During saccular and alveolar lung development, *Pdgfr*α^+^, *Shh*^+^, and *Fgf10*^+^ L-MSCs give rise to myofibroblasts and lipofibroblasts, which are crucial for alveolar development (119–122). Importantly, *Pdgfr*α + L-MSCs are supportive of lung epithelial progenitor cells, which are unable to form colonies in their absence or in the presence of more differentiated myofibroblasts (123, 124). There is mounting evidence from both human patients and animal models that L-MSCs are perturbed in BPD, potentially actively contributing to BPD pathogenesis. The presence of L-MSCs in tracheal aspirates from ventilated preterm infants could predict the subsequent development of BPD (125). *In vitro*, these L-MSCs showed signs of dysfunction through reduced PDGFRα expression, a propensity toward myofibroblast differentiation and impaired migration capacity (126, 127). This is supported by a recent study in neonatal mice, where suppression of *Fgf10* expression left alveolar epithelial type 2 cells (AEC2) unable to regenerate after hyperoxia damage, leading to increased AEC1 differentiation (128). Combined with prior observations in parabronchial smooth muscle cells upon naphthalene injury (129), the secretion of FGF10 to stimulate epithelial repair may be one of the ways through which L-MSCs exert their regenerative capacities in the distal lung following injury (130).

Similarly, lung resident ECFCs, which are important for the development of the pulmonary microvasculature, were shown to be dysfunctional in a neonatal rat model of BPD (131). Moreover, the cord blood of preterm infants who go on to develop BPD contains lower numbers of circulating ECFCs, which are more vulnerable to hyperoxia-induced oxidative stress and dysfunction (132). Understanding how these resident progenitor populations are affected in BPD, but also how they normally mediate development, repair, and regeneration in the lung, will provide an insight into how we may mobilize these cells to actively engage in repair and normalize lung development.

#### Potential Therapies

Tapping into and stimulating the regenerative properties of L-MSCs and ECFCs through cell-based therapy may be a central way to ameliorate the lung injury leading to BPD pathogenesis. To this end, important lessons will come from exogenous stem cell therapy. In a neonatal rat hyperoxia model of BPD, intratracheal installation of either bone marrow or umbilical cord derived MSCs, or their conditioned media, could nearly completely repair experimental BPD, both on a histological and on a functional level (133, 134). The mode of action appears to be largely paracrine, as injection with MSC conditioned medium could promote alternatively activated (M2) macrophages (135). Exosomes, which are extracellular vesicles containing a cocktail of proteins, RNAs and even mitochondria, are secreted by a wide variety of cells including MSCs and likely play an active role in the paracrine therapeutic effects of MSCs (136). Their potential as a carrier of therapeutic paracrine factors makes them appealing and promising targets for cell-free MSC based therapy. However, several technical challenges must be overcome to ensure their safety, such as a robust reproducible isolation technique and their ability to facilitate infectious or damaging particles (137). The next decade will likely see large advances in the development of exogenous stem cell therapy for BPD and a vast array of other diseases, either by injecting stem cells themselves, their conditioned medium or through exosomes [see recent reviews by Möbius and Thébaud (138), O’Reilly and Thébaud (139), and Mitsialis and Kourembanas (136)].

### Pulmonary Macrophages Contribute to Alveolar Development and Repair

Arguably the most important immune cells to participate in wound repair are alternatively activated macrophages. Besides peripheral blood derived macrophages, the pulmonary microenvironment contains three distinct resident pulmonary macrophage populations: alveolar macrophages, interstitial macrophages and primitive macrophages (140). Alveolar macrophages are the best-studied subset and are most abundantly present in the lung. They reside in the alveolar spaces where they phagocytose foreign particles and have a crucial role in the surfactant metabolism that facilitates alveolar function and gas exchange. Interstitial macrophages (IMF) reside on the other side of the epithelial barrier, among mesenchymal cells and capillaries. They have a distinct phenotype and behavior from alveolar macrophages and are geared more toward tissue repair and maintenance, antigen presentation and influencing dendritic cell functions to prevent allergy (140, 141). The third population, primitive macrophages, has only very recently been identified as a distinct subtype. These macrophages are the first to colonize the fetal lungs, and persist in adult lungs in the parenchyma of the peripheral alveoli. Because of their location in peripheral and perivascular spaces, which have been described as hotspots for alveolar regeneration, they are speculated to promote or be attracted to stem cell activity (140). The influx of these macrophages, which display an alternatively activated or M2 phenotype, and localization at the branching sites of the developing lung, suggest they potentially contribute to alveolar lung development (142). Conversely, if fetal lung macrophages are activated by an inflammatory stimulus, they actively inhibit expression of genes critical for lung development, leading to disrupted airway development and perinatal death in mice (143).

#### Potential Therapies

These insights provide new support for anti-inflammatory treatments. Furthermore, exogenous MSC therapy may be beneficial in regulating pulmonary macrophage activity. As cells with potent immunomodulatory capacities, MSCs can regulate macrophage function and polarization (144). Steady-state MSCs drive macrophages toward a wound healing or M2 phenotype through the production of IL-6 and inhibit differentiation toward dendritic cells (145, 146). However, in a proinflammatory environment MSCs stimulate macrophages toward a pro-inflammatory M1 phenotype (147). Using cell-based therapy to activate resident L-MSCs may therefore also be effective in promoting an M2 phenotype in pulmonary macrophages.

### The Lung Microbiome: An Important Emerging Field of Interest

Although there has been a surge in interest in the microbiome thanks to the Human Microbiome Project, the lung was not included in this research project. Research interest in the lung microbiome is now however on the rise, uncovering that not only the upper but also the lower airways are colonized, with numbers of 10–100 bacterial cells per 1,000 human cells being reported (148). The six most commonly detected bacterial phyla are found throughout the body, but composition varies per organ. In the lung, composition varies between different areas, making consistent sampling of the same area extremely important when comparing between groups. The lungs of newborn infants are already colonized at birth with a variety of bacterial phyla, most predominately *Acinetobacter* (149). The composition of the lung microbiome changes and stabilizes in the first month of life, but is decidedly different in lungs of children and adult patients with lung disease (148, 149). Interestingly, amniotic fluid and the placenta harbor their own microbiota, suggesting that fetal tissues already get colonized *in utero*, potentially having an effect on early immune cell maturation (148).

Inflammation frequently occurs in preterm infants, both antenatal (chorioamnionitis) and postnatal (sepsis), and can strongly perturb lung development (150). In the neonatal period, the immune system is still immature, and evidence is mounting that host-microbial interactions are necessary for development and homeostatic control of the immune system (151). Recently, a strong correlation was found between decreased diversity of the lung microbiome at the time of birth in preterm infants and the development of BPD (149, 152). Other studies correlated prolonged antibiotics use during the first week of life and BPD (153, 154). The protective effect of bacterial exposure in early life on asthma and allergy development, the “hygiene hypothesis,” is extensively studied, and a greater microbial diversity of commensal bacteria seems to underlie this protective effect (148). Beyond microbial diversity and exposure, the role of the lung microbiome in the regulation and maturation of the immature immune system and the developing neonatal lung is less clear. One route of how the lung microbiome might train the immature immune system is by inducing expression of programmed death ligand 1 (PD-L1) in pulmonary dendritic cells. Lack of microbial colonization, or blocking pulmonary PD-L1 during the first 2 weeks of life in mice, induced a disproportionate inflammatory response to allergens later in life (155).

An imbalanced microbiome, called dysbiosis, may further impact the inflammatory and tissue repair response to oxygen exposure, as beneficial bacteria are lost or overrun by other bacteria. An important emerging mechanism through which the microbiome can influence cell function is through the production of microbial metabolites, such as short chain fatty acids or tryptophane catabolites (156, 157). Tryptophane catabolites are produced via the enzyme indoleamine 2,3-dioxygenase 1 (IDO1) and function as agonists for the aryl hydrocarbon receptor (AhR). AhR activation leads to an immune suppressive response through the production of interleukin (IL) 22 and promotes development of regulatory T-cells (158). One genus of bacteria capable of metabolizing tryptophane into AhR agonists are Lactobacilli. The beneficial effects of tryptophane metabolites and Lactobacilli have been shown to inhibit inflammation and promote health in the gut, central nervous system and the lung (156, 157). Treatment of COPD patients with emphysema with the anti-inflammatory macrolide antibiotic azithromycin, resulted in increased levels of tryptophane catabolites in bronchoalveolar lavages, which decreased macrophage production of proinflammatory cytokines (156). In mice, intranasal administration of Lactobacilli was more potent in reducing allergic airway inflammation than intragastric administration, possibly linked to an increase in regulatory T lymphocytes in the lungs (159). Interestingly, Lactobacilli were found to be significantly less abundant in the lungs of preterm infants who develop BPD compared to preterm infants who are BPD resistant (152). Within this cohort, Lactobacilli abundance was particularly low in infants born to mothers with chorioamnionitis. Coincidentally, azithromycin treatment could reduce the risk of BPD in preterm infants (160), particularly those colonized with *Ureaplasma* spp., which have been associated with chorioamnionitis and BPD (161, 162). The beneficial impact of the lung microbiome and specifically Lactobacilli on lung development is supported by a study in mice, where there was a positive correlation between microbial abundance and lung development (163). Injection of Lactobacilli into the lungs of germ-free mice could improve alveolar development (163).

#### Potential Therapies

In the near future, a potentially interesting avenue of therapy for the prevention or treatment of BPD may be the further exploration of the benefits of azithromycin. Following the bacterial lung microbiome, the lung virome and mycobiome are now slowly also becoming unraveled, which may provide further insights and treatment opportunities (148, 164, 165). Additionally, the benefits of pre- or probiotics to promote a healthy growth promoting lung microbiome should be investigated, and in particular the presence of Lactobacilli. d-Tryptophane was recently identified as a potent probiotic that could ameliorate allergic airway inflammation in a mouse model of allergic airway disease, and may therefore also be of interest in the setting of BPD (166). One possible way to achieve the same effect as tryptophane catabolites may be through the proton pump inhibitor omeprazole, which induces detoxification enzyme cytochrome P540 (CYP)1A1 possibly through an AhR-mediated process (167). AhR signaling is protective against hyperoxic injury in human fetal pulmonary microvascular cells and neonatal mice, likely because of its potent effects on the gene expression of immunomodulatory and developmental pathways (168). Combined pre- and postnatal omeprazole administration could attenuate hyperoxic lung injury in preterm rabbits even at low doses, making omeprazole an interesting potential therapeutic intervention to prevent BPD (167). Further studies are needed to validate its effects and to ascertain that it has no adverse effects on other developing organs.

### Anti-inflammatory Agents

Bronchopulmonary dysplasia is primarily considered to be a developmental disease resulting from perinatal inflammation, and therefore specialists in the field have for the past decade called for a special focus on the development and improvement of anti-inflammatory therapies in BPD (169). Currently there are multiple anti-inflammatory therapies under investigation. Interleukin 1 receptor antagonist (IL1RA) is particularly promising, as it can prevent the development of experimental BPD when administered at a low dose in the neonatal rodent “double hit” model of BPD, consisting of hyperoxia and perinatal inflammation (170–172). In a sheep model for prenatal inflammation, intra-amniotic IL1RA could partially prevent the effects that lipopolysaccharide (LPS) had on lung maturation, measured as surfactant protein gene expression and lung compliance (173). Interestingly, preterm infants who go on to develop BPD have elevated levels of IL1RA in their tracheal aspirates (174). A more recent study in preterm ventilated baboon and human infants suggested however that an increased IL1β:IL1ra ratio on days 1 to 3 of life is more predictive of BPD (172). The same study provided compelling animal data that early IL1RA or glyburide therapy, which prevents the formation of the NLR family, pyrin domain containing 3 (NLRP3) inflammasome upstream of IL1β, can indeed ameliorate BPD development (172). IL1RA, also called anakinra or Kineret, and glyburide, also known as Diabeta, are both already approved by the Federal Drug Administration (FDA) for treatment in rheumatoid arthritis and type 2 diabetes, respectively, making them attractive treatment options. Future studies will have to show whether their use would also be safe in the neonatal setting.

Postnatal use of corticosteroids such as dexamethasone and hydrocortisone, which are potent anti-inflammatory compounds, can effectively reduce the incidence of BPD (175, 176). Despite this positive effect, there are significant adverse effects associated with systemic administration of corticosteroids. Short-term adverse effects include intestinal perforation, gastrointestinal bleeding, hypertension, hypertrophic cardiomyopathy, hyperglycemia, and growth failure, while follow-up studies pointed to adverse effects on neuronal development (175, 176). Experts in the field have therefore questioned whether the beneficial effects of reducing BPD and death can be weighed up to these significant adverse effects (175, 176), and are reluctant to recommend postnatal systemic corticosteroids for the prevention of BPD (177). A perhaps more compelling alternative would be to more specifically target the lung through intratracheal administration. Early results obtained with inhaled corticosteroids have been mixed (178, 179), likely due to its efficiency to reach the lung parenchyma. However, as more studies are being done, there is increasing evidence that inhaled corticosteroids prevent BPD and death when administered early, but long term follow-up studies are needed to assess the risk-benefit ratio (180–182). Recent *in vitro* studies in human fetal lungs attributed budenoside more potent anti-inflammatory effects than dexamethasone, swiftly decreasing gene expression of chemokines IL8 and CCL2 (MCP1) in whole lungs even in the presence of exogenous surfactant (183). Future validation studies should however closely monitor the combined effect of intratracheal corticosteroids and pre-existing pulmonary inflammation, as combined antenatal exposure of fetal sheep to LPS and corticosteroids had much stronger effects on lung inflammation and developmental pathways than either agent alone (184–187). Additionally, it will be important to validate with combined budenoside and surfactant treatment also has the potential to prevent BPD in premature infants that initially present with mild RDS and do not receive surfactant therapy (188).

#### Potential Therapies

As outlined above, IL1RA, glyburide, and inhaled budenoside are currently the most promising anti-inflammatory therapies that have the potential to prevent BPD in premature infants. However, more studies will have to look into the safety and potential long-term effects in human neonates.

### Reactive Oxygen Species (ROS) and Mitochondrial Dysfunction

Although BPD pathogenesis has a very multifactorial nature, with oxygen exposure, mechanical ventilation and inflammation as some of the most widely accepted causes, one common pathway is shared by these insults: the generation of ROS. In animal models, exposure of neonatal animals to hyperoxia within a specific time period is sufficient to induce a pathophysiology similar to BPD (189). Underlying this pathophysiology is an exaggerated mitochondrial oxidant stress in response in newborn mice compared to adults, with an overall lower expression of antioxidant enzymes (190). The response to hyperoxia is developmentally regulated, leading specifically to the production of mitochondrial ROS-dependent NADPH oxidase 1 (NOX1) expression in neonatal animals (191). Expression of antioxidant enzymes is controlled by AhR, as AhR-deficient fetal human pulmonary microvascular cells displayed significantly attenuated antioxidant enzyme expression and increased hyperoxic injury (192). Deficiency of another key antioxidant enzyme, extracellular superoxide dismutase (EC-SOD), was sufficient to impair alveolar development and induce pulmonary hypertension in mice (193). This phenotype was worsened by additional oxidative stress caused by bleomycin exposure, which was also associated with decreased VEGF signaling (193). Further support for the hypothesis that ROS formation also plays a role in human BPD development has come from a genetic study in very low birth weight infants, which found an association between single nucleotide polymorphisms (SNPs) in antioxidant response genes and an increased or decreased risk for the development of BPD (194). The role of antioxidant enzymes in neonatal chronic lung disease is reviewed in depth by Berkelhamer and Farrow (195).

Mitochondria play a central role in oxygen metabolism, and mitochondrial abundance as measured by mitochondrial protein expression peaks around birth to facilitate the transition to the oxygen-rich world outside the womb (196, 197). Preterm infants are born before this peak, making them less prepared to deal with this shift in bioenergetics. Besides this mitochondrial immaturity, the exposures leading to chronic lung diseases have been linked to mitochondrial dysfunction (198, 199). Both hyperoxia exposure and mechanical ventilation of neonatal mice caused pulmonary mitochondrial dysfunction (200, 201). Moreover, direct inhibition of mitochondrial oxidative phosphorylation significantly impaired alveolar development, comparable to hyperoxia or mechanical ventilation. *In vitro* experiments indicate that elevated CO_2_ levels, called hypercapnia, a common occurrence in BPD patients, also causes mitochondrial dysfunction (202). One potential mechanism through which mitochondrial dysfunction and ROS generation potentially lead to impaired alveolar development in hyperoxia exposed neonatal mice is through endoplasmic reticulum (ER) stress, which can cause apoptosis (82).

#### Potential Therapies

In animal studies, several potential treatments have been identified to decrease ROS generation. In neonatal mice, treatment with a specific mitochondrial antioxidant, (2-(2,2,6,6-tetramethylpiperidin-1-oxyl-4-ylamino)-2-oxoethyl)triphenylphosphonium chloride (mitoTEMPO), could protect against hyperoxia-induced lung injury (191). Another promising treatment compound is GYY4137, a slow-releasing H_2_S donor, which could decrease ROS generation and thus protect and restore normal alveolar and microvascular development after neonatal hyperoxia injury in rats (203). Targeting the AhR would appear to be another promising approach considering it also has potent anti-inflammatory properties, as described above. Although omeprazole is generally seen as a potentiator of AhR activation, omeprazole treatment of hyperoxia-exposed newborn mice counterintuitively decreased functional AhR activation, worsening hyperoxic injury (204). Other approaches to promote AhR activation may however prove to be more effective. An entirely different approach in treating mitochondrial dysfunction may be through mitochondrial transfer, a process that has been reported as one of the therapeutic mechanisms of MSC therapy (205). In human BPD patients, most neonatal antioxidant trials have unfortunately not shown any benefit in the prevention of BPD, with the exception of vitamin A therapy (195). However, none of these antioxidant therapies were specifically targeted against mitochondrial ROS or dysfunction. More targeted approaches, as those outlined in the animal studies, may prove to be more promising.

### Other Promising Therapeutic Options Based on Novel Pathophysiological Insights

Inflammation associated with BPD pathogenesis affects many molecular pathways, which by themselves can be interesting therapeutic targets. One of these is the ceramide pathway, which is upregulated in both hyperoxia and antenatal inflammation animal models (206–208) and also in other chronic lung diseases such as asthma, cystic fibrosis and COPD (209). Increased ceramide levels lead to increased apoptosis, both in epithelial cells of BPD patients and in animal models of BPD (208, 209). Intervention with a sphingosine-1-phosphate (S1P) analog in the mouse hyperoxia model of BPD could successfully ameliorate ceramide levels and hyperoxia-induced alveolar hypoplasia (208). In a more complex piglet model of lung injury by lavage, LPS instillation and injurious ventilation, tracheal installation with surfactant and d-myo-inositol-1,2,6-trisphosphate (IP3) could achieve a similar effect in reducing ceramide levels and improving oxygenation (210). In a different approach to decrease sensitivity to apoptosis in hyperoxia-exposed epithelial cells, inhibiting regulatory-associated protein of mechanistic target of rapamycin (RPTOR) could prevent hyperoxia-induced lung injury in neonatal mice (211). Based on these studies, selective pharmacological interventions which temporarily reduce apoptosis could be a promising way to prevent or repair neonatal lung injury and reduce BPD severity.

An intervention that has garnered attention in neonatal care is lactoferrin (LF), an iron-binding protein that is a normal component of human colostrum and milk (212). It has potent antimicrobial activity, can stimulate the innate immune system and promote epithelial proliferation and differentiation of the immature gut (213). Recent studies have identified LF supplementation as a promising agent for the reduction of late onset sepsis and necrotizing enterocolitis (214). Although the properties of LF may also be desirable for the prevention of BPD, to date no study has been able to show a significant reduction in the development of BPD following LF supplementation (214).

A pathophysiological mechanism of BPD that is slowly gaining more attention is the link between pre-eclampsia and BPD. Pre-eclampsia a proven risk factor for BPD (41), and the underlying impact on the developing fetus may be three-fold. Firstly, maternal preeclampsia is a frequent cause of preterm birth before 28 weeks (215). Secondly, severe preeclampsia can lead to intrauterine growth restriction, which in itself is a strong risk factor for BPD (38, 39). Thirdly, the placental dysfunction that lies at the root of pre-eclampsia leads to an overproduction of soluble VEGF receptor 1 [also known as soluble fms-like tyrosine kinase-1 (sFlt-1)], which inhibits VEGF signaling (216, 217). This not only leads to increased sVEGFR-1 in maternal serum, but also in amniotic fluid (218). By giving pregnant rats intra-amniotic injections with sVEGFR-1, Steven Abman’s group demonstrated a link between pre-eclampsia and BPD, as neonatal rats presented with impaired alveolar and microvascular development and right and left ventricular hypertrophy (40). Moreover, intrauterine exposure to excess sVEGFR-1 led to increased apoptosis of endothelial and mesenchymal cells in neonatal rat lungs. Placental dysfunction and subsequent overexpression of sVEGFR-1 may therefore be a potential therapeutic target to improve fetal outcome and prevent development of BPD. At the very least, the diagnosis of maternal pre-eclampsia should be considered as a serious predisposition for the development of BPD.

From a developmental biology perspective, developmental molecular pathways that are downregulated in BPD provide other potential targets for the amelioration of BPD pathogenesis. These include the Wnt signaling pathway (187, 219, 220), SHH signaling (185, 221–223), axonal guidance cues semaphorin 3 C and ephrin B2 (224, 225), Notch signaling (226, 227), and HIFs (228). In addition, a wealth of new molecular insights on mouse and human lung development has been and will be published in the upcoming years by the LungMAP consortium (1U01HL122638), funded by the National Heart, Lung, and Blood Institute (NHLBI) (http://www.lungmap.net) (229, 230). BPD is generally considered to be caused by environmental factors, but in recent years studies have uncovered that a genetic component may also be at play [reviewed in Ref (231, 232)]. Although associations are not conclusive, these studies suggest that genetic variants of genes in well-known lung development and repair pathways may predispose for severe BPD or mild/moderate BPD (232). microRNAs have emerged as both a pathophysiological mechanism and a tempting tool to target transcription of multiple of these developmental signaling pathways at once. Although multiple human and animal studies have reported an association between altered microRNA levels and BPD, valid concerns have been raised about the lack of a causal link between altered microRNA levels and BPD pathogenesis [reviewed in Ref (233)]. However, if such a causal link can be confirmed, as was recently seen in a study which demonstrated the regulation of alveolar septation by microRNA-489 (234), the use of specific microRNA antagonists or agonists may be considered as a potential therapy for BPD. Caution should however be exercised when directly modulating potent developmental pathways, either directly or through microRNA therapy. Further exploration of such therapeutic targets should perhaps be combined with slow releasing microparticles or capsules to ensure a more physiological release and prevent pathological side effects.

### Conclusion and Future Directions

**Figure 3:**
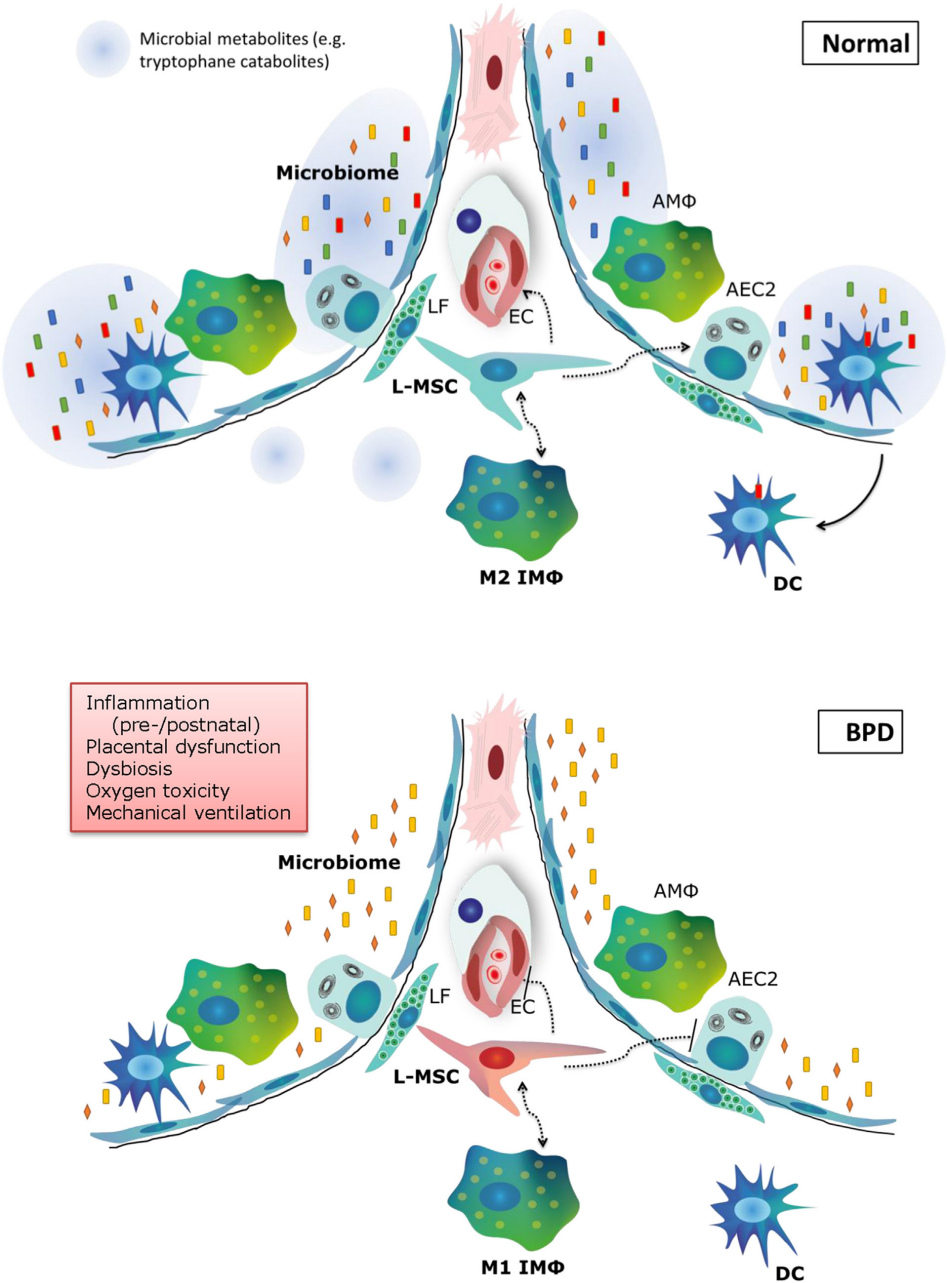
**Summary of new pathophysiological concepts in bronchopulmonary dysplasia (BPD)**. In normal alveolar lung development, a diverse microbiome is necessary to train the pulmonary immune system and secrete metabolites that support lung development. Pulmonary M2 interstitial macrophages (M2 IMφ) are present and likely play an active role in lung development. L-MSCs support M2 IMφ, alveolar epithelial cells and the microvasculature. In BPD (bottom panel), pre- and postnatal risk factors lead to decreased microbiome diversity, a proinflammatory environment, dysfunctional L-MSCs, epithelial and endothelial injury and impaired repair. LF, lipofibroblast; EC, endothelial cell; AMΦ, alveolar macrophage; L-MSC, lung mesenchymal stromal cell; AEC2, alveolar epithelial cell type 2; M1/M2 IMΦ, type 1/2 interstitial macrophage; DC, dendritic cell.

The pathophysiology of BPD is extremely multifactorial, which is underlined by the emerging role of cell types that have only recently been acknowledged, such as the microbiome, macrophages, and tissue stem cells (Figure 3). Our knowledge on the pathophysiology is poised to move forward rapidly in the next decade, due to exciting new technological advances in the research field, and is opening avenues for the pursuit of therapeutic options. In addition, there is still promise for new and better applications of existing therapies, which have not yet fulfilled their promise in a clinical setting. In the next decade of BPD research, the most promising therapies and pathophysiological concepts that should be pursued for new therapeutic options are as follows:

- Animal models investigating the pathogenesis of BPD should identify different sub-pathophysiological processes that arise because of different combinations of pre-and postnatal exposures (e.g. pre-eclampsia, dysbiosis), as opposed to only looking at hyperoxia or inflammation models. Moreover, better appreciation of extrapulmonary issues related to BPD might be instructive, particularly neurodevelopmental outcome and retinopathy, which are frequent long-term outcomes resulting from BPD (235).
- Different routes of administration for effective therapies such as vitamin A and postnatal corticosteroids, in particular non-invasive intratracheal routes.
- Cell-based therapies, either through administration of stem cells and their products or by promoting the regenerative potential of resident lung stem cells.
- The commensal role of the pre- and postnatal (lung) microbiome in the normal and perturbed lung development, and its potential as a therapeutic target.
- The role of placental dysfunction in the pathogenesis of BPD, and its potential as a therapeutic target in the prevention of BPD.
- The role of the immune system not only as an adverse factor in BPD pathogenesis, but its importance in supporting normal lung development and repair.

## Author Contributions

Conception and outline of review: JC, DT, and RR. Writing of the manuscript: JC. Drafting of the manuscript: JC, DT, and RR. Critical revision of manuscript: JC, DT, IK, IR, and RR. Final approval of manuscript: JC, DT, IK, IR, and RR.

## Conflict of Interest Statement

The authors declare that the research was conducted in the absence of any commercial or financial relationships that could be construed as a potential conflict of interest.
